# Characteristics of the vaginal microbiota and vaginal metabolites in women with cervical dysplasia

**DOI:** 10.3389/fcimb.2024.1457216

**Published:** 2024-10-10

**Authors:** Tiantian Yu, Shan Gao, Fen Jin, Bingbing Yan, Wendong Wang, Zhongmin Wang

**Affiliations:** ^1^ Dalian Medical University, Dalian, China; ^2^ Female Pelvic Floor Urinary Reconstructive Center, Dalian Women and Children’s Medical Group, Dalian, China; ^3^ Department of Engineering Mechanics, Dalian University of Technology, Dalian, China

**Keywords:** cervical cancer, cervical lesions progression, vaginal microbiota, vaginal metabolites, correlation analysis

## Abstract

**Introduction:**

Emerging evidence suggests that the vaginal microbiota is closely associated with cervical cancer. However, little is known about the relationships among the vaginal microbiota, vaginal metabolites, and cervical lesion progression in women undergoing cervical dysplasia.

**Methods:**

In this study, to understand vaginal microbiota signatures and vaginal metabolite changes in women with cervical lesions of different grades and cancer, individuals with normal or cervical dysplasia were recruited and divided into healthy controls (HC) group, low-grade squamous intraepithelial lesions (LSIL) group, high-grade squamous intraepithelial lesions (HSIL) group, and cervical cancer (CC) group. Vaginal secretion samples were collected for 16S rRNA gene sequencing, liquid chromatography coupled with mass spectrometry (LC–MS)-based metabolomics, and integrated analysis.

**Results:**

The results demonstrated that bacterial richness and diversity were greater in the CC group than the other three groups. Additionally, *Lactobacillus* was found to be negatively associated with bacterial diversity and bacterial metabolic functions, which increased with the degree of cervical lesions and cancer. Metabolomic analysis revealed that distinct metabolites were enriched in these metabolite pathways, including tryptophan metabolism, retinol metabolism, glutathione metabolism, alanine, aspartate, and glutamate metabolism, as well as citrate cycle (TCA cycle). Correlation analysis revealed positive associations between CC group-decreased *Lactobacillus* abundance and CC group-decreased metabolites. *Lactobacillus iners* was both negative to *nadB* and *kynU* genes, the predicted abundance of which was significantly higher in the CC group. The linear regression model showed that the combination of the vaginal microbiota and vaginal metabolites has good diagnostic performance for cervical cancer.

**Discussion:**

Our results indicated a clear difference in the vaginal microbiota and vaginal metabolites of women with cervical dysplasia. Specifically altered bacteria and metabolites were closely associated with the degree of cervical lesions and cancer, indicating the potential of the vaginal microbiota and vaginal metabolites as modifiable factors and therapeutic targets for preventing cervical cancer.

## Introduction

Cervical cancer has the highest incidence of female reproductive system malignant tumor, most common in 40–60-year-old women, and in recent years, the incidence has been increasing progressively (Al Saedi et al., 2022; Benitez-Paez et al., 2020). According to literature reports in 2022, there were about 150,700 new cases in China, and the number of deaths due to cervical cancer was 55,700, accounting for about one-third of the cases (Zheng et al., 2024).

Persistent infection with high-risk human papillomavirus (HPV) has been shown to be a necessary factor for the development of cervical squamous intraepithelial lesion (SIL) and cervical cancer (Perkins et al., 2023). However, it cannot completely explain the pathogenesis of cervical precancerous lesions and cervical cancer. At present, many scholars have found that vaginal microbiota played a certain role in the occurrence of genital HPV infection, cervical lesions, and even cervical cancer (Ilhan et al., 2019; Xu et al., 2022; Zhang et al., 2024). The human vaginal microbiota is different from the gut microbiota; its composition and diversity are simple. *Lactobacillus* is the normal dominant bacterial group of female vaginas and plays a key role in maintaining the normal ecological balance of vaginas (Kovachev, 2020). It produces a large amount of lactic acid by decomposing the glycogen of vaginal epithelial cells, which is conducive to maintaining the acidic environment in the vagina, secreting hydrogen peroxide (H_2_O_2_), bacteriocins, and biosurfactants, stimulating the immune defense of body, protecting the vagina from infection by pathogens and other abnormal colonizing microorganisms, and maintaining the “self-purification effect” of the vagina (Fan et al., 2021). Studies have shown that those subjects with *Lactobacillus* as the dominant bacteria were more conducive to the reversal of SIL; meanwhile, the decrease of *Lactobacillus* and pathogenic bacteria colonization are related to the progression of SIL (Mitra et al., 2020).

Cancer is a metabolic disease. Studies have shown that different metabolites produced by different bacteria may have pathogenic or protective effects, and vaginal microecological metabolites may transfer through the host barrier to affect the systemic health of high-risk cervical cancer patients (Wishart et al., 2016). Related studies have shown that the metabolism of amino acids, peptides, and nucleotides in the vagina of patients with cervical lesions was changed (Ilhan et al., 2019). It was found that lipid and organic acid metabolism was changed in patients with cervical cancer (Xu et al., 2022). There were significant differences in the metabolism of biogenic amines, glutathione, and lipids between HPV-positive and HPV-negative women in vaginal metabolome (Borgogna et al., 2020). A strong correlation between changes in vaginal microbiota and serum metabolism during HPV infection was reported—for example, α-linolenic acid and lactic acid bacteria are strongly associated in HPV infection progression (Zhang et al., 2024).

Vaginal metabolism can represent the characteristics of the vaginal microenvironment and host response; meanwhile, integrating multiple omics data may be necessary to achieve better performance for cervical lesion prediction (Subramanian et al., 2020). The combination of vaginal microbiota and vaginal metabolism can more specifically reflect the changes of the vaginal microenvironment in cervical lesion progression. It is essential to clarify the specific mechanisms involved in the interaction between the vaginal microbiota, vaginal metabolism, and cervical lesion progression. The correlation will provide potential directions to explore the mechanism and potential early diagnostic indicators of cervical cancer and lead to the use of novel interventions to improve the level of female health. To the best of our knowledge, so little literatures reported this research topic so far.

In this study, we characterized the vaginal microbiota profile and signatures of vaginal metabolites in women with various degrees of cervical lesions. This project used an integrated approach combining 16S rRNA gene sequencing with LC–MS-based metabolomics analysis of vaginal secretion to determine whether specific vaginal microbiota and their metabolites are associated with cervical cancer progression. In this study, we evaluated the characteristics of vaginal microbiota, vaginal metabolites, and their interactions in women with cervical lesions, thus providing new insights into the pathogenesis and therapeutic targets of cervical cancer.

## Materials and methods

### Study subjects and sample collection

A total of 121 patients diagnosed with cervical lesions for the first time and patients found normal at physical examination were collected from Dalian Women and Children’s Medical Group, including 30 healthy controls (HC group), 29 patients with LSIL (LSIL group), 33 patients with HSIL (HSIL group), and 29 patients with CC (CC group). Basic clinical information was collected, including age, number of miscarriages, number of births, height, weight, and body mass index (BMI). Detailed clinical information is presented in **Supplementary Table S1**.

This study was approved by the Ethics Committee of Dalian Women and Children’s Medical Group (approval number: FEJT-KY-2024-52). The inclusion criteria were that all patients with SIL and CC had been pathologically confirmed and had not yet received relevant treatment. Subjects who had no sexual life history, menstruating or pregnant, used antibiotics and antifungal drugs within 3 months, had vaginal irrigation within 1 week or had sex within 48 h, or other conditions (such as hypertension, diabetes, hepatitis B, tuberculosis, and immune system diseases) were excluded. Vaginal secretion was collected from each subject and transported to an -80°C freezer for further analysis.

### Microbiota 16S rRNA gene sequencing

Total genome DNA from vaginal secretion samples was extracted using CTAB/SDS method. DNA concentration and purity was monitored on 1% agarose gels. According to the concentration, DNA was diluted to l μg/μL using sterile water. The same volume of IX loading buffer (containing SYB green) was mixed with PCR products, and electrophoresis was operated on 2% agarose gel for detection. The PCR products were mixed in equidensity ratios. Then, the mixture of PCR products was purified with Qiagen Gel Extraction Kit (Qiagen, Germany). Vaginal secretion DNA was isolated, and the V3/V4 hyper-variable regions of the bacterial 16S rRNA gene was amplified by primers 341F (CTACGGGNGGCWGCAG) and 805R (GACTACHVGGGTATCTAATCC). After denaturation for 1 min at 98°C, 30 cycles were performed, each lasting 10 s, followed by annealing at 50°C for 30 s and elongation at 72°C for 30 s. As soon as the PCR products were purified, TruSeq DNA PCR-free sample preparation kits (Illumina, USA) and index codes were added to create sequencing libraries. Lastly, the libraries were sequenced on an Illumina NovaSeq platform, and 250-bp paired-end reads were generated. The QIIME V.2.0 pipeline was used after barcodes were taken off the sequences (Hall and Beiko, 2018). Random selection of reads was used to reduce the amplicon sequence variants (ASVs) of each sample to 10,000 (Valles-Colomer et al., 2022). The ASV taxonomy was based on the Silva 138 SSURef NR99 16S rRNA gene reference database (Quast et al., 2013; Zhao et al., 2022).

### Analysis of vaginal microbiota profile

The alpha diversity of vaginal microbiota was estimated by using R package Vegan, and the beta diversity was estimated via the vegdist function of Vegan R package based on the genus levels’ Bray–Curtis distance matrix (Lloyd-Price et al., 2019). The significant difference of the beta diversity was statistic by PERMANOVA test (adonis function). The bacterial composition was analyzed by using phyloseq R package (McMurdie and Holmes, 2013). Furthermore, LEfSe (linear discriminant analysis effect size) analysis was performed via the microbiomeMarker R package (Cao et al., 2022). Moreover, the predicted function of microbiota was estimated by using PICRUSt2 (Phylogenetic Investigation of Communities by Reconstruction of Unobserved States 2) (Douglas et al., 2020).

### Metabolite extraction and LC–MS analyses

Three swabs containing vaginal secretion were placed in Eppendorf tubes, and 1 mL prechilled 80% methanol was added. After well vortex and sonification on ice for 5 min, they were centrifuged at 15,000 *g* at 4°C for 20 min. The supernatant was freeze-dried and dissolved with 10% methanol. Finally, the solution was injected into the LC–MS system for analysis.

LC–MS analyses were performed using a Vanquish UHPLC system (Thermo Fisher, Germany) coupled with an Orbitrap Q Exactive™ HF mass spectrometer (Thermo Fisher, Germany). Samples were injected onto a Hypersil Goldcolumn (100*2.1 mm, 1.9 μm) using a 12-min linear gradient at a flow rate of 0.2 mL/min. The eluents for the positive polarity mode were eluent A (0.1% FA in water) and eluent B (methanol). The eluents for the negative polarity mode were eluent A (5 mM ammonium acetate, pH 9.0) and eluent B (methanol). The solvent gradient was set as follows: 2% B, 1.5 min; 2%–85% B, 3 min; 85%–100% B, 10 min; 100%–2% B, 10.1 min; 2% B, 12 min. Q Exactive™ HF mass spectrometer was operated in positive/negative polarity mode with spray voltage of 3.5 kV, capillary temperature of 320 °C, sheath gas flow rate of 35 psi, and aux gas flow rate of 10 L/min, S-lens RF level of 60, and aux gas heater temperature of 350°C.

### Data processing and metabolite identification

The raw data generated by LC–MS were processed using the Compound Discoverer 3.3 software to perform peak alignment, peak picking, and quantitation for each metabolite. The main parameters were set as follows: peak area was corrected with the first QC, actual mass tolerance, 5 ppm; signal intensity tolerance, 30%; and minimum intensity. After that, peak intensities were normalized to the total spectral intensity. The normalized data was used to predict the molecular formula based on additive ions, molecular ion peaks, and fragment ions. Then, the peaks were matched with the mzCloud, mzVault, and MassList database to obtain accurate qualitative and relative quantitative results. Statistical analyses were performed using the statistical software R (version 3.4.3), Python (version 2.7.6), and CentOS (version 6.6). When data were not normally distributed, these were standardized according to the formula: sample raw quantitation value (the sum of sample metabolite quantitation value/the sum of QC1 sample metabolite quantitation value) to obtain relative peak areas. Compounds whose CVs of relative peak areas in QC samples were greater than 30% were removed, and finally the metabolites’ identification and relative quantification results were obtained.

### Analysis of vaginal secretion metabolomics

A difference in vaginal metabolite signatures was detected by the gradient progression of cervical lesions among different groups using LC–MS. For the identification of metabolites, processed data, such as mass-to-charge ratio (*m*/*z*), retention time (RT), and normalized peak areas, were imported into SIMCA. Metabolites were identified using the HMDB (Human Metabolome Database). The HMDB database was adopted to map and identify the metabolites. By using R package ropls to identify the metabolites’ changes within groups, partial least squares discriminant analysis (PLS-DA) was used to analyze the abundance of significant metabolites with projection (VIP) >1 and *p* value (Wilcoxon test) < 0.05. The enrichment pathway with gradient progression of cervical lesions among HC, LSIL, HSIL, and CC vaginal metabolites’ profiles was analyzed by using MetaboAnalyst 5.0 (Pang et al., 2021). The differentiation between host and vaginal bacteria-derived metabolites was analyzed by using MetOrigin (Yu et al., 2022).

### Correlation analysis

Using Spearman’s correlation analysis, the correlation between predicted function and changed microbiota, changed microbiota, and metabolites was analyzed. R package “psych”, version 2.4.1, was used in the correlation analysis.

### Marker panel for CC individuals

In the discovery dataset, a linear regression model was built based on the signatures of microbiota or metabolites of CC subjects (Liang et al., 2020). The possibility of the model was estimated by predicted function in both discovery and validation sets. Besides this, the accuracy of the marker panel to discriminate the CC participants from HC, LSIL, and HSIL cases in both discovery and validation sets was employed by using R package pROC (Robin et al., 2011).

### Statistical analysis

All statistical analysis and graphical representations of the study were performed via R. Data were expressed in mean ± SEM unless otherwise stated. We utilized Wilcoxon and chi-square tests to evaluate the difference between different groups for continuous and categorical variables. The significant difference between different groups was at a confidence level of 0.05.

## Results

### Characteristics of the vaginal microbiota profile

**Figure 1:**
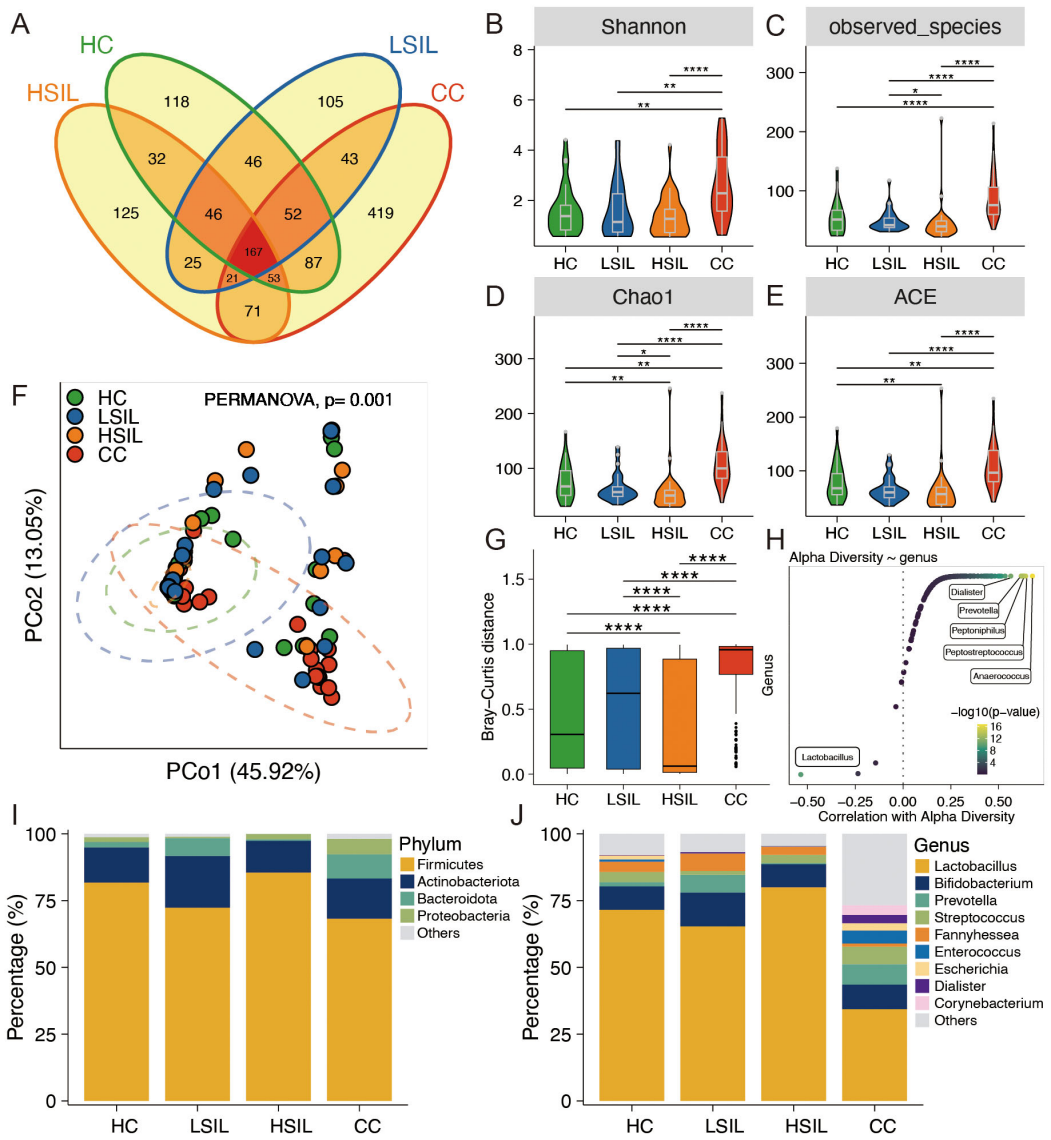
Characteristics of the vaginal microbiota composition. **(A)** Petal diagram exhibition for four groups. **(B–E)** Alpha diversity comparison of microbiota among four groups. **(F)** PCoA analysis of vaginal microbiota at the genus level. **(G)** Bray–Curtis distance among four groups. **(H)** Correlation between bacteria at the genus level with alpha diversity. **(I, J)** Vaginal microbiota composition in four groups at the phylum and genus levels, respectively. *p<0.05, **p<0.01, ****p<0.0001.

The petal diagram, constructed based on the number of ASVs, revealed that the CC group exhibited the highest diversity with respect to different ASVs, while the HC, LSIL, and HSIL groups displayed relatively similar numbers (**Figure 1A**). This diagram visually demonstrated both similarities and differences in ASV flora among these four groups. Furthermore, various diversity indices including Shannon, observed species, chao1, and ACE were calculated to assess the vaginal microbiota in each group. The results indicated a significantly higher diversity of vaginal microbiota in the CC group compared to the other three groups (**Figures 1B-E**). In addition, we employed principal coordinate analysis (PCoA) to observe the dissimilarities among sample groups. The PCoA of the genus abundance of the vaginal microbiota revealed clear differences between the CC group and the other three groups (**Figure 1F**). In the CC group, the Bray–Curtis distance of the samples was higher than the other three group, indicating a diversity between samples; intra-group was highest in the CC group (**Figure 1G**). As shown in **Figure 1H**, *Lactobacillus* showed a negative correlation with α-diversity. Meanwhile, *Dialister*, *Prevotella*, *Peptoniphilus*, *Peptostreptococcus*, and *Anaerococcus* were positively correlated with α-diversity (**Figure 1H**). At the phylum level, major vaginal bacteria included *Firmicutes*, *Actinobacteria*, *Bacteroidetes*, and *Proteobacteria* (**Figure 1I**). At the genus level, major vaginal bacteria included *Lactobacillus*, *Bifidobacterium*, *Prevotella*, *Streptococcus*, *Fannyhessea*, *Enterococcus*, *Escherichia*, *Dialister*, and *Corynebacterium* (**Figure 1J**).

### Identification of the signatures of the vaginal microbiota profile and metabolic function

**Figure 2:**
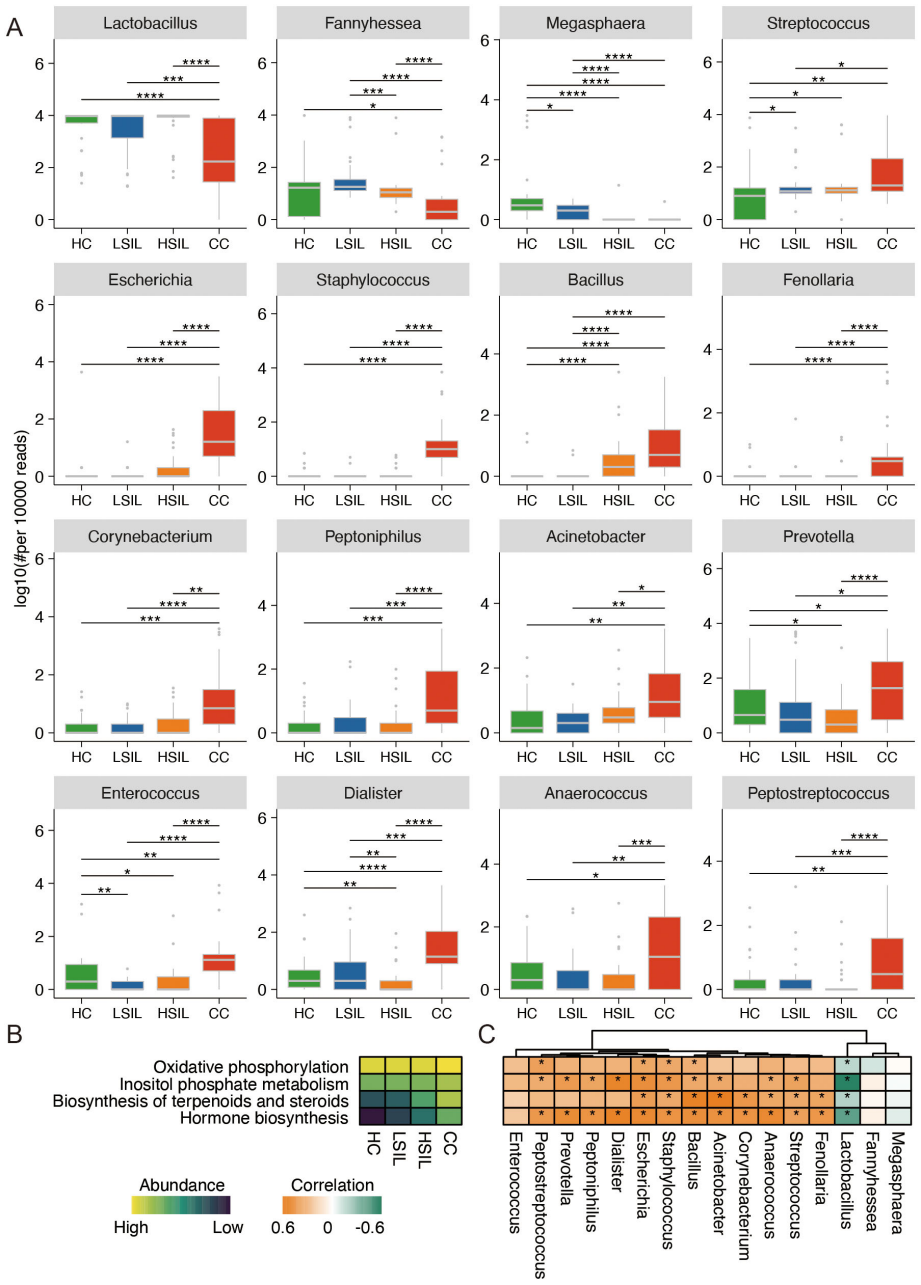
Characteristic difference and function of vaginal microbiota. **(A)** Significant difference of vaginal microbiota at the genus level. **(B)** Remarkable difference in predicted function of bacteria among four groups. **(C)** Correlation analysis of predicted function and bacteria abundance. *p<0.05, **p<0.01, ***p<0.001, ****p<0.0001.

At the genus level, *Lactobacillus*, *Fannyhessea*, and *Megasphaera* showed a descending gradient abundance in the CC group in comparison with the other three groups, along with disease progression. The abundance of *Streptococcus*, *Escherichia*, *Staphylococcus*, *Bacillus*, *Fenollaria*, *Corynebacterium*, *Peptoniphilus*, and *Acinetobacter* was ascending with the degrees of cervical lesions and cancer. Other significantly changed bacteria, such as *Prevotella*, *Enterococcus*, *Dialister*, *Anaerococcus*, and *Peptostreptococcus*, did not show a gradient change with the degrees of cervical lesions and cancer (**Figure 2A**).

The results of metabolic function prediction analysis showed that the abundance of oxidative phosphorylation, inositol phosphate metabolism, biosynthesis of terpenoids and steroids, and hormone biosynthesis showed a progressive increase with cervical cancer progression in the four groups of samples (**Figure 2B**). *Lactobacillus* was negatively correlated with the four types of metabolic function. *Bacillus*, *Staphylococcus*, and *Escherichia* were positively correlated with four types of metabolic function (**Figure 2C**).

### The vaginal metabolome revealed a distinct metabolism in cervical lesion progression

**Figure 3:**
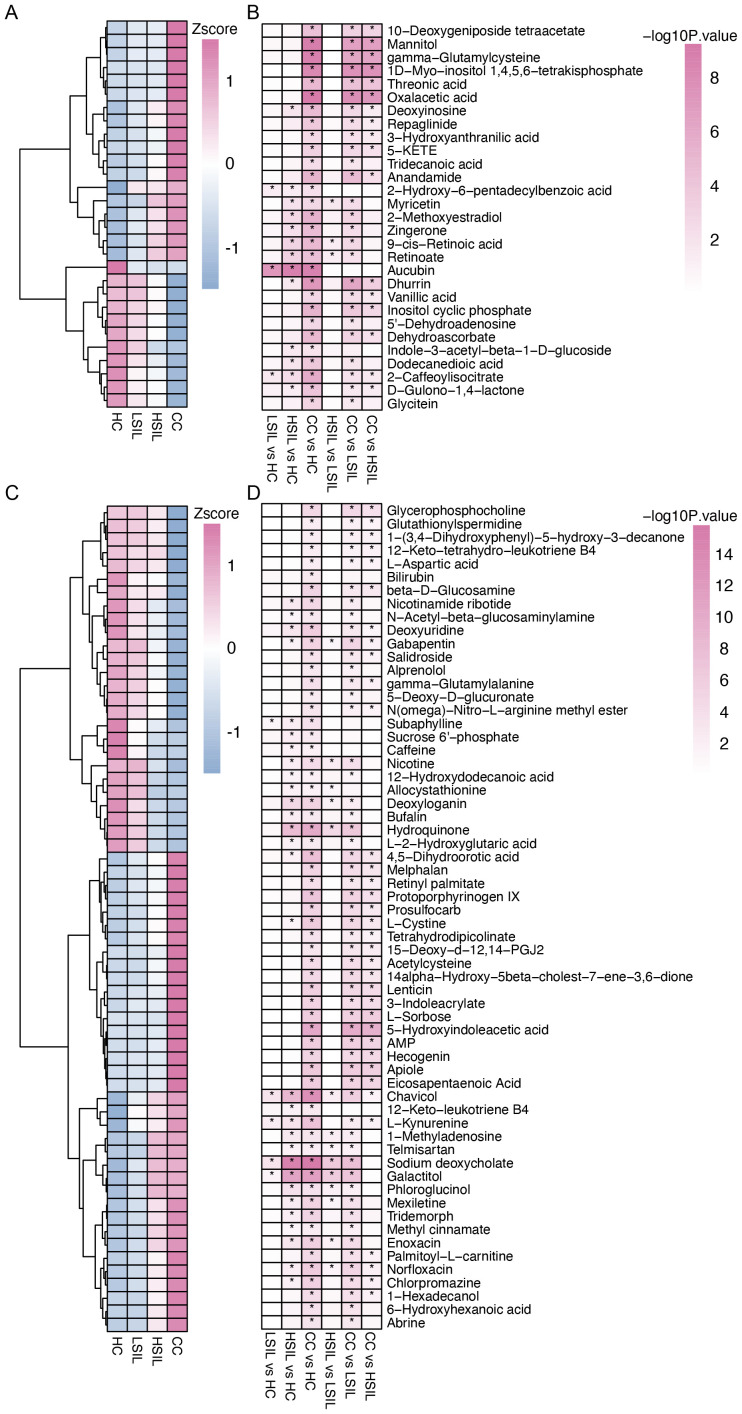
Characteristic difference of vaginal metabolites. **(A, B)** Changed metabolites with cervical lesion progression and comparison between each two groups in the NIM. **(C, D)** Changed metabolites with cervical lesion progression and comparison between each two groups in the PIM. *p<0.05.

We further analyzed vaginal metabolites by using LC–MS, focusing on negative ion mode (NIM) and positive ion mode (PIM). In the NIM, the levels of 18 metabolites exhibited an increase, and 11 metabolites exhibited a decrease with the degrees of cervical lesions and cancer (**Figures 3A, B**). In the PIM, the levels of 36 metabolites exhibited an increase, and 26 metabolites exhibited a decrease (**Figures 3C, D**). A comparative analysis was conducted for each two groups in the four groups.

**Figure 4:**
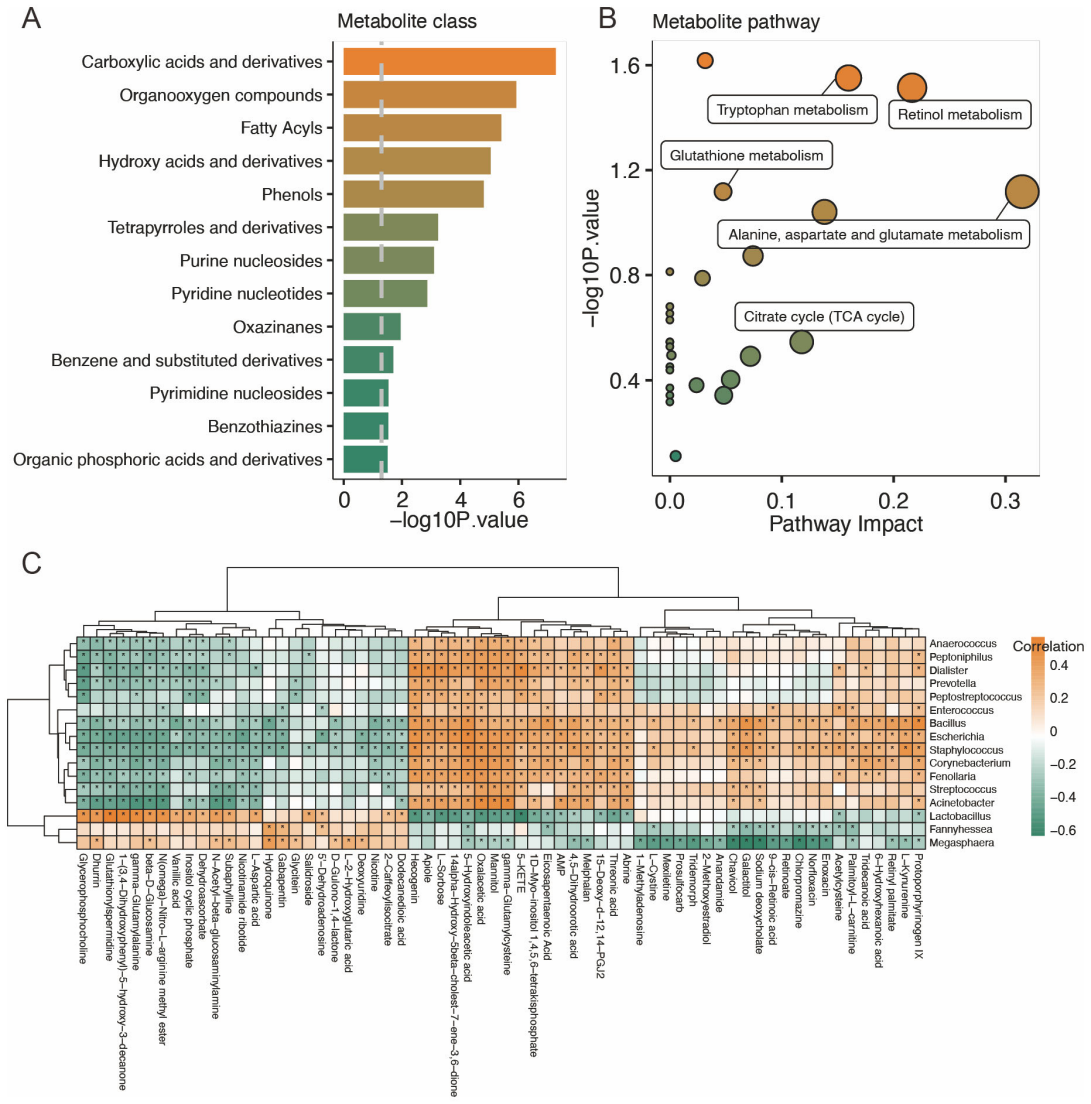
Characteristics of the vaginal metabolites and correlation with vaginal microbiota. **(A, B)** Metabolites’ sets and KEGG pathway enrichment based on different metabolites. **(C)** Correlation analysis of distinct vaginal microbiota and vaginal metabolites. *p<0.05.

The results of the metabolite classes showed that the altered metabolites mainly belonged to carboxylic acid and derivatives, organooxygen compounds, fatty acyls, hydroxy acids and derivatives, and phenols (**Figure 4A**). Distinct metabolites were enriched in the following metabolite pathways: tryptophan metabolism, retinol metabolism, glutathione metabolism, alanine, aspartate, and glutamate metabolism, as well as TCA cycle (**Figure 4B**).

### Association analysis of the vaginal microbiota and vaginal metabolites

A correlation analysis was performed to study the relationship between the distinct vaginal microbiota and vaginal metabolites. The results showed that *Lactobacillus* is positively correlated with dodecanedioic acid, 2-caffeoylisocitrate, 5′-dehydroadenosine, salidroside, L-aspartic acid, subaphylline, n-acetyl-beta-glucosaminylamine, dehydroascorbate, inositol cyclic phosphate, vanillic acid, n(omega)-nitro-L-arginine methyl ester, beta-D-glucosamine, gamma-glutamylalanine, 1-(3,4-dihydroxyphenyl)-5-hydroxy-3-decanone, glutathionylspermidine, glutathionylspermidine, dhurrin, and glycerophosphocholine and negatively correlated with protoporphyrinogen IX, acetylcysteine, abrine, threonic acid, 15-deoxy-d-12,14-PGJ2, 4,5-dihydroorotic acid, AMP, eicosapentaenoic acid, 1D-myo-inositol 1,4,5,6-tetrakisphosphate, 5-KETE, gamma-glutamylcysteine, mannitol, oxalacetic acid, 5-hydroxyindoleacetic acid, 14alpha-hydroxy-5beta-cholest-7-ene-3,6-dione, L-sorbose, apiole, and hecogenin (**Figure 4C**). Other correlations between distinct bacteria and metabolites are also displayed.

### Integrated analysis of the host- and vaginal bacteria-derived vaginal metabolites

**Figure 5:**
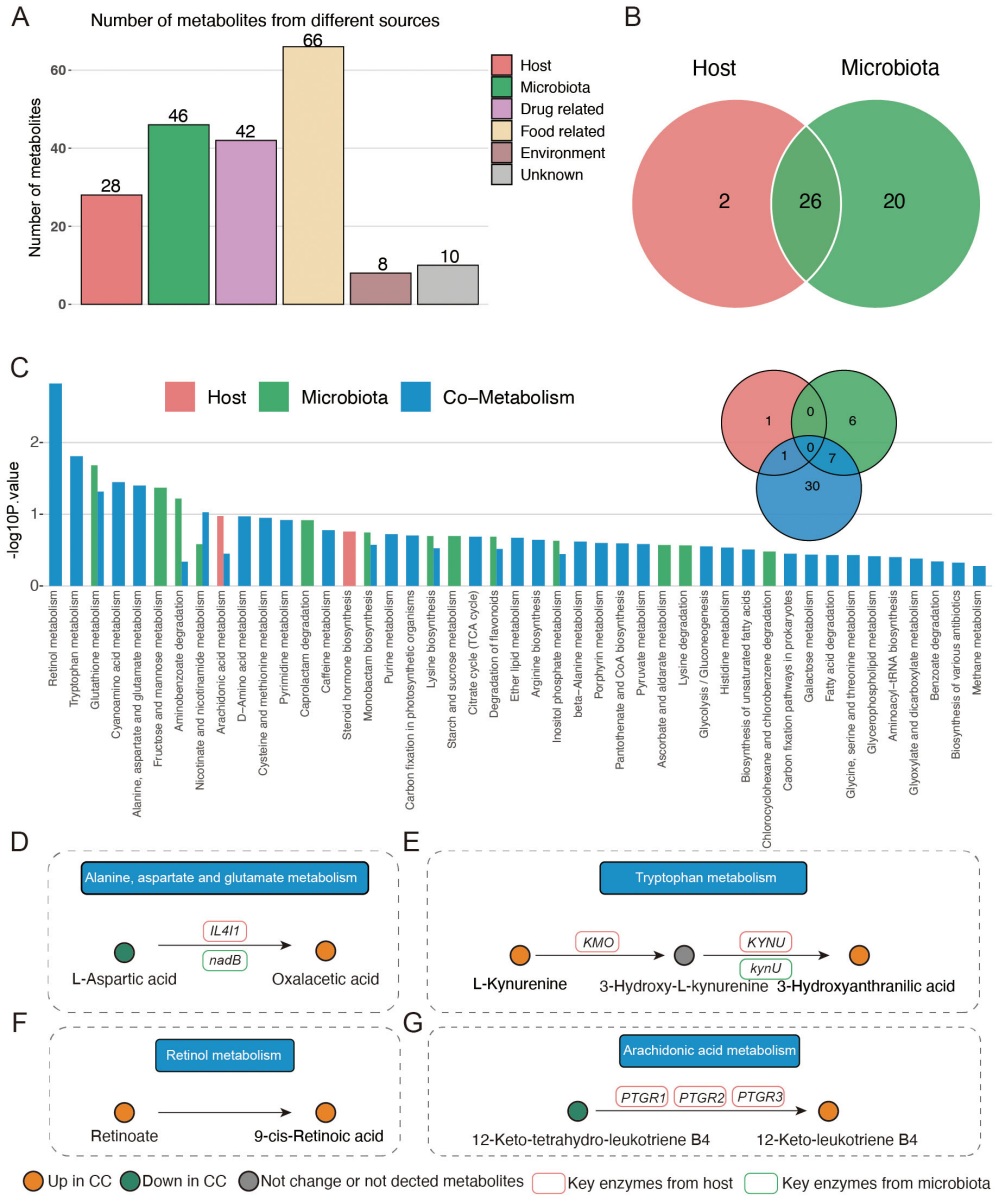
Origin analysis of vaginal metabolites. **(A)** Source of different vaginal metabolites among four groups. **(B)** Venn plot indicating the number of metabolites originating from host and vaginal microbiota. **(C)** Pathway enrichment based on host- and vaginal-microbiota-derived metabolites. **(D–G)** Illustration of significantly enriched pathways.

Next, we analyzed the origins of the vaginal metabolites that had significant gradient changes with the degrees of cervical lesions and cancer. As shown in **Figure 5A**, 28 and 46 vaginal metabolites were identified as originating from the host and vaginal microbiota, respectively. Among them, 26 metabolites were both derived from host and vaginal microbiota (**Figure 5B**). A pathway enrichment analysis of metabolites suggested that alanine, aspartate, and glutamate metabolism, tryptophan metabolism, and retinol metabolism occurred via co-metabolism by the host and vaginal microbiota. Arachidonic acid metabolism was mainly metabolized by the host and partly by co-metabolism (**Figure 5C**). In alanine, aspartate, and glutamate metabolism, vaginal bacteria *nadB* gene and host *IL4I1* gene facilitate the production of oxaloacetic acid from L-aspartic (**Figure 5D**). The vaginal bacteria *kynU* gene, as well as host *KMO* and *KYNU* genes, generally catalyzes the production of 3-hydroxyanthranilic acid (**Figure 5E**). The host *PTGR1*, *PTGR2*, and *PTGR3* genes promote the conversion of 12-keto-tetrahydro-leukotriene B4 to 12-keto-leukotriene B4 in arachidonic acid metabolism (**Figure 5F**). Retinol metabolism showed no association with key enzymes from host or microbiota (**Figure 5G**). In addition, oxaloacetic acid, 3-hydroxyanthranilic acid, and 12-keto-leukotriene B4 were all produced in the CC group.

### Combined biomarkers to discriminate cervical cancer patients

**Figure 6:**
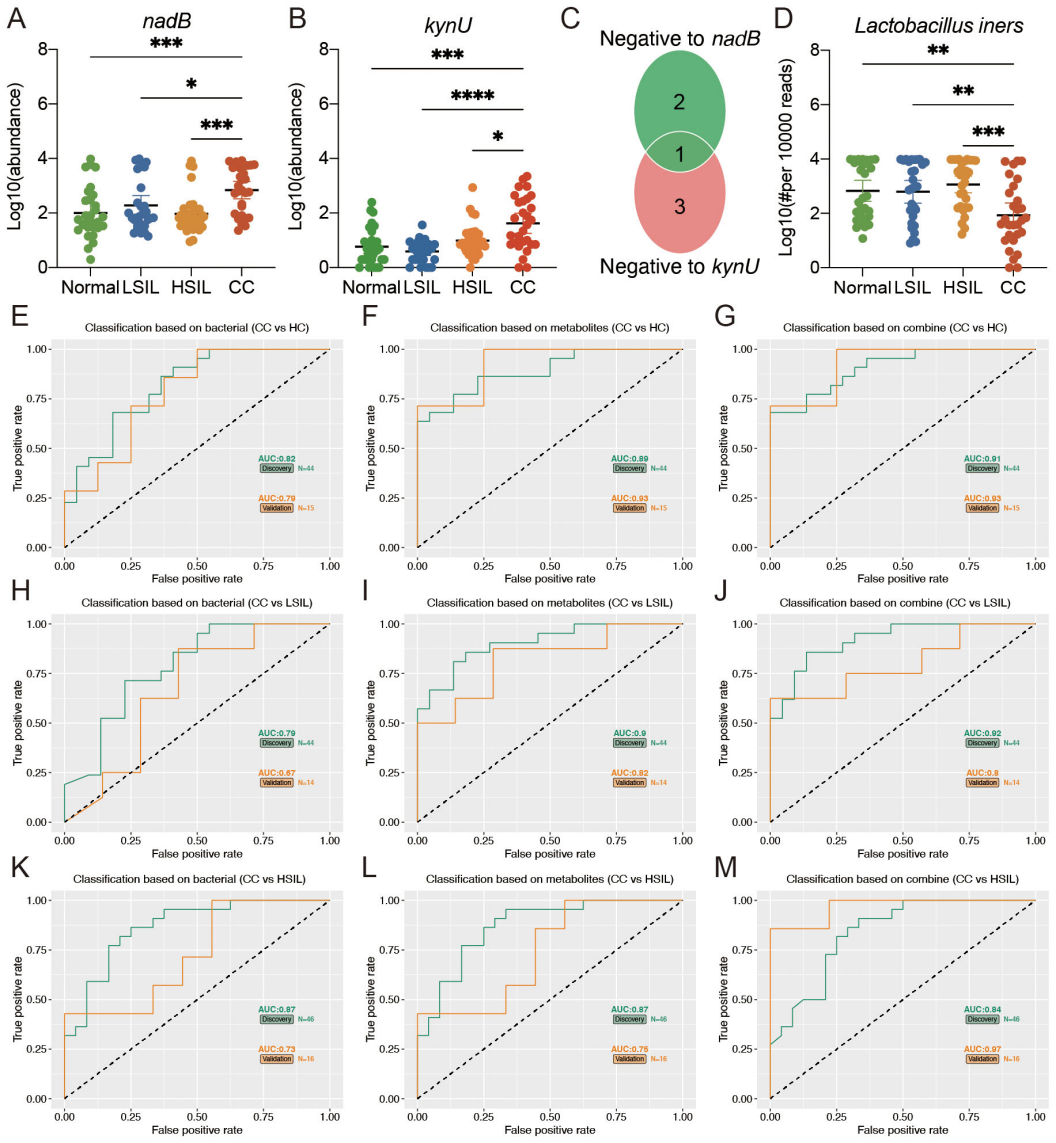
Multiple markers for the diagnosis of cervical cancer. **(A, B)** Predicted vaginal microbiota *nadB* and *kynU* genes’ abundance based on PICRUTS2. **(C)** The Venn plot indicated that the bacteria belonging to *Lactobacillus* at the species level was negative to *nadB* and *kynU* genes. **(D)** Comparison of *Lactobacillus iners* among four groups. **(E, F, H, I, K, L)** Separated vaginal microbiota and vaginal metabolites diagnose CC from HC, LSIL, and HSIL. **(G, J, M)** Accuracy of the combined marker panels to diagnose CC from other groups. *p<0.05, **p<0.01, ***p<0.001, ****p<0.0001.

The predicted abundance of the *nadB* and *kynU* genes in the vaginal microbiota of the CC group was significantly higher than that of the other three groups (**Figures 6A, B**). In addition, the *Lactobacillus* genus was strongly correlated with the *nadB* and *kynU* genes. We further compared the abundance of vaginal microbiota that belong to the *Lactobacillus* genus at the species level. As shown in **Figures 6C, D**, the abundance of *Lactobacillus iners*, which was negative to both *nadB* and *kynU* genes, significantly decreased in the CC group than in the other three groups.

We further constructed a linear regression model based on the CC signature-related vaginal microbiota (*Lactobacillus iners*) and vaginal metabolites (oxalacetic acid and 3-hydroxyanthranilic acid) to predict cervical cancer. As shown in **Figures 6E-I, K, L**, the individual marker panels could discriminate CC individuals from HC, LSIL, and HSIL in both the discovery and validation sets. Importantly, the combination of the vaginal microbiota and vaginal metabolites has good diagnostic performance (**Figures 6G, J, M**).

## Discussion

In healthy women, the vaginal microbiota maintains a dynamic equilibrium with anti-infection capabilities, effectively hindering adherence and proliferation of exogenous substances and pathogens (Integrative, 2019). Hormonal changes, age, sexual practices, and antimicrobial drug use can influence the composition and diversity of the vaginal microbiota (Hickey et al., 2015; Mulder et al., 2019; Plummer et al., 2019; Zapata and Quagliarello, 2015), causing the dysfunction of the vaginal microbiota. The microbial dysbiosis increases the likelihood of exogenous pathogen infection, including HPV (Buchta, 2018). A persistent HPV infection has the potential to trigger cervical precancerous lesions and eventually cervical cancer. However, less is known about the vaginal microbiota and metabolite signatures in women with cervical lesions of different grades and cancer. Cervical cancer progression is a slow disease process (Koliopoulos et al., 2017), but the increasing incidence of cervical cancer has led to a significant burden for patients and society. It is essential to find ideal targets for researching the risk factors and developing cervical cancer for intervention and treatment in advance. Here, for the first time, we characterized the vaginal microbiota profile and signatures of vaginal metabolites in women with cervical lesions of different grades and cancer. Our results indicated a clear difference in the vaginal bacterial composition and vaginal metabolite abundance among the HC, LSIL, HSIL, and CC groups. The specific alterations in the vaginal microbiome and derived metabolites were strongly associated with the degrees of cervical lesions and cancer, indicating the potential of the vaginal microbiota and vaginal metabolites as modifiable factors and therapeutic targets for preventing cervical cancer.

The results of this study showed a significant increase in the diversity of vaginal microbiota in the CC group, which was consistent with previous reports (Klein et al., 2019; Xu et al., 2022). Meanwhile, no significant alterations were observed in the diversity of LSIL and HSIL. Previous studies indicated that an increase in vaginal microbiota diversity was associated with HPV infection rather than the status of cervical lesions, thus supporting our findings (Chen et al., 2020). *Lactobacillus* is the main probiotic in the normal vaginal microbiota, which plays a key role in maintaining the normal ecological balance of the vagina. It can decompose the glycogen of vaginal epithelial cells to produce lactic acid, maintain the acidic environment in the vagina, and secrete H_2_O_2_, bacteriocins, and biosurfactants to prevent the invasion of pathogenic bacteria (Di Paola et al., 2017; Stoyancheva et al., 2014). In this study, with the degrees of cervical lesions and cancer, the abundance of *Lactobacillus* genus showed a gradient decline, and the results of a related study showed that *Lactobacillus*, as the dominant bacterial group in the vagina, was more conducive to the reversal of SIL (Mitra et al., 2020), which further verified the results of our study. In our study, *Lactobacillus* was negatively correlated with α-diversity, which was reported for the first time, further confirming the main probiotic effect of *Lactobacillus* in the vagina. Consistent with our findings, pernicious bacteria, such as *Streptococcus*, *Escherichia*, *Staphylococcus*, *Bacillus*, *Corynebacterium*, *Peptoniphilus*, and *Acinetobacter*, increased with the degrees of cervical lesions and cancer in this study and were also closely related to cervical cancer (Chen et al., 2020; Jiang et al., 2021; Klein et al., 2019; Manzanares-Leal et al., 2022; Peremykina et al., 2024; Zou et al., 2024).

In this study, we also performed a functional predictive analysis of vaginal microbiota. The results showed that the pathways involved in oxidative phosphorylation, inositol phosphate metabolism, biosynthesis of terpenoids and steroids, and hormone biosynthesis were enhanced with the degrees of cervical lesions and cancer. Due to the uncontrolled proliferation of cancer cells, the energy produced by aerobic glycolysis appears to be insufficient to support cellular metabolism during cell proliferation. Therefore, cellular metabolism in cell proliferation provides energy through the oxidative phosphorylation pathway (Tango et al., 2020). Previous studies have reported that oxidative phosphorylation was increased in breast cancer (Miko et al., 2019); hence, the high metabolic demand could explain the enhanced enrichment of oxidative phosphorylation pathway in the degrees of cervical lesions and cancer. The research reported that inositol polyphosphate phosphatase 1 (INPP1), as a key enzyme in inositol phosphate metabolism, could drive cancer aggressiveness by reducing glycolysis and lipid metabolism, which was consistent with the results of our study (Benjamin et al., 2014). A further correlation analysis of the vaginal microbiota and metabolic pathways revealed that the abovementioned metabolic pathways were negatively correlated with *Lactobacillus*, which was decreased with the degrees of cervical lesions and cancer. Moreover, *Escherichia*, *Staphylococcus*, and *Bacillus*, which were increased with the degrees of cervical lesions and cancer, showed a positive correlation with the above-mentioned pathways. These results indicate that some vaginal bacteria are closely associated with cervical cancer, but further studies are needed to determine the underlying mechanisms involved in cervical cancer.

Moreover, vaginal secretion metabolism analysis demonstrated that the levels of 18 metabolites exhibited an increase and 11 metabolites exhibited a decrease with the degrees of cervical lesions and cancer in the NIM, and the levels of 36 metabolites exhibited an increase and 26 metabolites exhibited a decrease in the PIM. Furthermore, an association analysis of the vaginal microbiota and vaginal metabolites revealed descending levels of dodecanedioic acid, 2-caffeoylisocitrate, 5′-dehydroadenosine, salidroside, L-aspartic acid, subaphylline, n-acetyl-beta-glucosaminylamine, dehydroascorbate, inositol cyclic phosphate, vanillic acid, n(omega)-nitro-L-arginine methyl ester, beta-D-glucosamine, gamma-glutamylalanine, 1-(3,4-dihydroxyphenyl)-5-hydroxy-3-decanone, glutathionylspermidine, glutathionylspermidine, dhurrin, and glycerophosphocholine with the degrees of cervical lesions and cancer, showing a positive correlation with *Lactobacillus*. The ascending levels of protoporphyrinogen IX, acetylcysteine, abrine, threonic acid, 15-deoxy-d-12,14-PGJ2, 4,5-dihydroorotic acid, adenosine monophosphate (AMP), eicosapentaenoic acid, 1D-myo-inositol 1,4,5,6-tetrakisphosphate, 5-KETE, gamma-glutamylcysteine, mannitol, oxalacetic acid, 5-hydroxyindoleacetic acid, 14alpha-hydroxy-5beta-cholest-7-ene-3,6-dione, L-sorbose, apiole, and hecogenin with the degrees of cervical lesions and cancer were negatively correlated with *Lactobacillus*. In addition, the altered metabolites were mainly enriched in carboxylic acid and derivatives, organooxygen compounds, fatty acyls, hydroxy acids and derivatives, and phenols. Furthermore, the enrichment of relevant metabolic pathways that differed significantly with the degrees of cervical lesions and cancer, based on differentially abundant metabolites, indicated the enrichment of tryptophan metabolism, retinol metabolism, glutathione metabolism, alanine, aspartate and glutamate metabolism, and TCA cycle. The anticancer properties of salidroside on breast, ovarian, cervical, colorectal, lung, liver, gastric, bladder, renal, and skin cancer as well as gliomas and fibrosarcomas have previously been demonstrated (Sun and Ju, 2021), and the present study confirms these findings. L-aspartic acid exhibited inhibitory activity and antiproliferative activity against the cervical cancer cell lines (Han et al., 2017), which is in agreement with our findings. The most potent dioxin, 2,3,7,8-tetrachlorodibenzo-p-dioxin (TCDD), caused vacuolization of the smooth endoplasmic reticulum and Golgi apparatus in cultured human conjunctival epithelial cells and cervical cancer cells, and dehydroascorbic acid can protect against TCDD-induced cell damage (Hirai et al., 2002). The phytophenolics from *Caesalpinia mimosoides Lamk*, which contained vanillic acid, showed cytotoxic effects on cervical carcinoma cell lines through an apoptotic pathway (Palasap et al., 2014). The anticancer activity of glucosamine conjugation to zinc(II) complexes of a bis-pyrazole ligand on human breast adenocarcinoma, human cervical cancer, and human lung adenocarcinoma was verified, consistent with our results (Bhattacharyya et al., 2014). Alloimperatorin was recently reported to induce autophagy in cervical cancer cells via the reactive oxygen species pathway. However, N-acetylcysteine reversed the autophagy, which is in agreement with our results (yingBai et al., 2022). Inhibition of the major cyclic adenosine monophosphate (AMP)-metabolizing enzyme PDE4 has shown a potential for the discovery of drugs for cancer, inflammation, and neurodegenerative disorders such as Alzheimer’s disease. The results confirmed the roles of AMP in cervical cancer (Quimque et al., 2021). Inconsistent with our study, previous studies showed that eicosapentaenoic acid had selective tumoricidal action (Das and Madhavi, 2011). Therefore, detailed research for the effect of eicosapentaenoic acid on cervical cancer is needed. To the best of our knowledge, the other metabolites above had not been reported to be associated with cervical cancer. In terms of metabolic pathways, tryptophan metabolism in the progression of cervical cancer has been investigated in numerous studies (Hascitha et al., 2016; Qu et al., 2023), and tryptophan metabolism could become an important target for developing new pharmacological treatments for cervical cancer. Recent research showed that alcohol dehydrogenase 7 (ADH7), which was enriched in the retinoic acid metabolic process and the retinol metabolism pathway, played pivotal roles in the progression of cervical cancer and was significantly associated with cervical cancer survival rate (Ding et al., 2020). The relationship between glutathione metabolism and cervical cancer has long been established. Relevant research has indicated that alterations in erythrocyte glutathione metabolism were associated with cervical dysplasia and carcinoma *in situ*. Meanwhile, the changes in erythrocyte glutathione-related indices in conjunction with histopathological diagnosis may have the potential to distinguish between low- and high-grade cervical dysplastic lesions (Basu et al., 1993). Alanine, aspartate, and glutamate metabolism as well as TCA cycle had not been demonstrated to be related to cervical cancer.

Among the mentioned metabolic pathways, alanine, aspartate, and glutamate metabolism, tryptophan metabolism, and retinol metabolism were co-metabolized by host and vaginal microbiota, and arachidonic acid metabolism was mainly metabolized by host and partly by co-metabolism. Moreover, the predicted abundance of vaginal microbiota *nadB* and *kynU* genes, which were involved in the mentioned metabolic pathways, in the CC group was significantly higher than that in the other groups. *Lactobacillus* genus was strongly correlated with *nadB* and *kynU* genes. By comparing the abundances of vaginal microbiota that belong to the *Lactobacillus* genus at the species level, we found that only the abundance of *Lactobacillus iners* was negative to both *nadB* and *kynU* genes. *Lactobacillus iners* has been reported in numerous studies to be related to cervical cancer. However, to the best of our knowledge, *nadB* and *kynU* genes have never been reported to be associated with cervical cancer, indicating that further studies are needed to investigate the potential action and underlying mechanisms of these genes on cervical cancer.

In this study, we also focused on the early diagnostic value of vaginal microbiota or vaginal metabolites for cervical cancer. Using the linear regression model, we confirmed that the presence of vaginal microbiota combined with vaginal metabolites could be a better indicator for predicting cervical cancer. A linear regression model was recently used as a practical method for the early diagnosis of cervical lesions after screening with multiple feature selection methods (Nieves-Ramirez et al., 2021). Individual marker panels of vaginal microbiota or vaginal metabolites could discriminate CC individuals from HC, LSIL, and HSIL individuals in both the discovery and validation sets. Importantly, the combination of vaginal microbiota and vaginal metabolites had a greater diagnostic performance. Cervical cancer has the highest incidence of female reproductive system malignant tumor, most commonly in 40–60-year-old women. In recent years, the incidence has been increasing progressively. Therefore, it is highly important to effectively predict the occurrence of cervical lesions at an early stage.

## Conclusion

In conclusion, our results suggested that women with cervical lesions of different grades and cancer have significant changes in vaginal microbiota and vaginal metabolites. This correlation provides potential directions for exploring the mechanism and potential early diagnostic indicators of cervical cancer. This study might lead to the use of novel interventions to improve the level of female health.

## Data Availability

The data presented in the study are deposited in the NCBI repository, accession number PRJNA1159925.
