# Corrigendum: Characteristics of the vaginal microbiota and vaginal metabolites in women with cervical dysplasia

**DOI:** 10.3389/fcimb.2024.1527287

**Published:** 2024-11-19

**Authors:** Tiantian Yu, Shan Gao, Fen Jin, Bingbing Yan, Wendong Wang, Zhongmin Wang

**Affiliations:** ^1^ Dalian Medical University, Dalian, China; ^2^ Female Pelvic Floor Urinary Reconstructive Center, Dalian Women and Children’s Medical Group, Dalian, China; ^3^ Department of Engineering Mechanics, Dalian University of Technology, Dalian, China

**Keywords:** cervical cancer, cervical lesions progression, vaginal microbiota, vaginal metabolites, correlation analysis

In the published article, there was an error regarding the affiliations for Tiantian Yu, Zhongmin Wang. As well as having affiliations 1, they should also have “Dalian Medical University, Dalian, China”.

The authors apologize for this error and state that this does not change the scientific conclusions of the article in any way. The original article has been updated.

