# Supplementary material for: Characteristics of the vaginal microbiota and vaginal metabolites in women with cervical dysplasia
Source: Front Cell Infect Microbiol. 2024 Oct 10;14:1457216. doi: 10.3389/fcimb.2024.1457216 (PMC11499233; doi:10.3389/fcimb.2024.1457216)
Supplement: Supplementary file 1 [file Table1.docx]

**Table S1.** Clinical characteristics of subjects in each group

| **Characteristics** | HC group (n=30) | LSIL group (n=29) | HSIL group (n=33) | CC group (n=29) |
| --- | --- | --- | --- | --- |
| **Age (years)** | 38.07±0.558 | 37.69±5.114 | 38.00±6.514 | 49.89±11.390 |
| **Height (cm)** | 163.72±4.209 | 163.36±5.387 | 162.88±4.878 | 163.14±4.964 |
| **Weight (kg)** | 59.17±8.568 | 59.03±9.590 | 61.85±8.243 | 63.91±13.207 |
| **BMI** | 22.04±2.716 | 22.07±3.262 | 23.33±3.100 | 23.94±4.430 |
| **Number of births** | 0.97±0.490 | 0.93±0.651 | 0.82±0.528 | 1.32±1.056 |
| **Number of miscarriages** | 0.63±0.718 | 1.21±1.013 | 1.15±0.972 | 1.64±1.545 |

Values are mean ± SD for continuous variables.
